# Teneligliptin Exerts Antinociceptive Effects in Rat Model of Partial Sciatic Nerve Transection Induced Neuropathic Pain

**DOI:** 10.3390/antiox10091438

**Published:** 2021-09-09

**Authors:** Yaswanth Kuthati, Vaikar Navakanth Rao, Prabhakar Busa, Chih-Shung Wong

**Affiliations:** 1 Department of Anesthesiology, Cathy General Hospital, Taipei 280, Taiwan; yaswanthk1987@gmail.com (Y.K.); Prabhakar.busa01@gmail.com (P.B.); 2 Department of Biomedical Sciences, Academia Sinica Institute, Taipei 11529, Taiwan; kanthuind89@gmail.com; 3 National Defense Medical Center, Institute of Medical Sciences, Taipei 280, Taiwan

**Keywords:** neuropathic pain, partial sciatic nerve transection, dipeptidyl peptidase-4 inhibitor, teneligliptin, glucagon-like peptide 1, glial fibrillary acidic protein

## Abstract

Neuropathic pain (NP), is a chronic pain resulting from nerve injury, with limited treatment options. Teneligliptin (TEN) is a dipeptidyl peptidase-4 inhibitor (DPP-4i) approved to treat type 2 diabetes. DPP-4is prevent the degradation of the incretin hormone glucagon-like peptide 1 (GLP-1) and prolong its circulation. Apart from glycemic control, GLP-1 is known to have antinociceptive and anti-inflammatory effects. Herein, we investigated the antinociceptive properties of TEN on acute pain, and partial sciatic nerve transection (PSNT)-induced NP in *Wistar* rats. Seven days post PSNT, allodynia and hyperalgesia were confirmed as NP, and intrathecal (i.t) catheters were implanted and connected to an osmotic pump for the vehicle (1 μL/h) or TEN (5 μg/1 μL/h) or TEN (5 μg) + GLP-1R antagonist Exendin-3 (9–39) amide (EXE) 0.1 μg/1 μL/h infusion. The tail-flick response, mechanical allodynia, and thermal hyperalgesia were measured for 7 more days. On day 14, the dorsal horn was harvested and used for Western blotting and immunofluorescence assays. The results showed that TEN had mild antinociceptive effects against acute pain but remarkable analgesic effects against NP. Furthermore, co-infusion of GLP-1R antagonist EXE with TEN partially reversed allodynia but not tail-flick latency. Immunofluorescence examination of the spinal cord revealed that TEN decreased the immunoreactivity of glial fibrillary acidic protein (GFAP). Taken together, our findings suggest that TEN is efficient in attenuation of PSNT-induced NP. Hence, the pleiotropic effects of TEN open a new avenue for NP management.

## 1. Introduction

NP results from the damage or injury to the nerves in the peripheral or central nervous system (CNS). The common causes for NP include spinal cord injury (SCI), ischemic disease, viral disease, and alcoholism [1]. NP is commonly accompanied by allodynia and hyperalgesia. NP significantly decreases the quality of life by interfering with mobility and sleep. Currently, available treatment options include the use of antidepressants, anticonvulsants, and anti-epileptics. These medications are often combined with opioids to increase the analgesic efficacy in clinical practice [2,3,4,5,6]. Though these medications are effective against acute pain, they have limited efficacy against NP, and prolonged use can often lead to tolerance, severe side effects, and addiction [7]. Thus, there is an urgent need for new therapeutics for the management of NP.

Recent studies suggest that DPP-4i have valuable pleiotropic effects that can alleviate kidney, brain, and heart damage from oxidative stress [8,9,10]. TEN is relatively a new DPP-4i with pleiotropic effects and has a distinct chemical structure and pharmacokinetic profile when compared to other anti-diabetic therapeutics in the same category [11,12]. TEN is recently approved for the management of type-2 diabetes mellitus and is known to be safe in patients with renal and heart problems with a human plasma half-life of 24 h [13]. Apart from DPP-4 inhibition, oral administration of TEN is shown to have moderate analgesic effects in human subjects against thermal pain [14]. TEN has also displayed various other pleiotropic properties. For example, treatment with TEN is reported to greatly improve endothelial dysfunction by enhancing endothelium-derived nitric oxide synthase (NOS) in an animal model of metabolic syndrome [15]. It is also shown to prevent atherosclerosis through the suppression of monocyte chemoattractant protein-1 expression, and pro-inflammatory cytokine production. In addition, TEN is also known to enhance glutathione antioxidant production within the cells [16]. Indeed, TEN is reported to possess more ·OH scavenging ability compared to the naturally occurring antioxidant glutathione and to possess direct ·OH scavenging ability [17]. These properties are not connected to DPP-4 inhibition as TEN has displayed free radical scavenging properties in DPP-4-deficient animals [17]. Interestingly, other DPP-is in the same class did not exhibit O^2−^ or OH scavenging properties, making TEN an attractive compound for further research in neurological diseases [17].

Recent evidence suggests that TEN enhances the positive effects of GLP-1 [18]. Several previous studies have concluded that activation of GLP-1R can effectively suppress neuroinflammation and central sensitization, thereby alleviating neuropathic pain in various animal models [19,20,21,22,23,24,25]. Furthermore, in the CNS, GLP-1R is confined to microglial cells of the spinal dorsal horn and greatly decreased after nerve injury and has been found to play a key role in microglial cell activation [19,26]. Exenatide, a GLP-1R agonist, is effective in the alleviation of oxaliplatin-induced peripheral neuropathy in rats [27]. Exenatide’s analgesic properties were reversed by GLP-1 antagonists and GLP-1 gene knockdown [27]. Exendin-4, a peptide agonist of GLP-1 attenuated pain-induced cognitive impairment through the alleviation of neuroinflammation in NP rats [28]. In addition, DPP-4i like sitagliptin and GLP-1 analog liraglutide are shown to induce axonal regrowth and locomotor functional repair through the restoration of spinal GLP-1R levels in SCI rats [29,30].

Herein, we hypothesized that the TEN’s pleiotropic effects in the spinal dorsal horn play a beneficial role in offering protection against neuroinflammation in NP. Therefore, in the present study, we examined the antinociceptive and neuroprotective effects of TEN in acute pain and PSNT-induced NP. 

## 2. Materials and Methods

### 2.1. Reagents

TEN was obtained from MCE (Princeton, NJ, USA). EXE and naloxone were purchased from Tocris Bioscience (Ellisville, MS, USA). MOR and Dimethyl sulfoxide (DMSO) were obtained from Sigma-Aldrich (St. Louis, MO, USA). TEN, naloxone, and MOR were prepared in 4% DMSO. The saline-DMSO and other drugs used in osmotic pumps had an equal concentration of DMSO, to eliminate the solvent effect. A similar concentration of solvent did not show any analgesic effects in previous reports [31,32].

### 2.2. Animals

The protocols followed in the current experiments were evaluated and permitted by the animal use committee of the National Defense Medical Center, Taipei, Taiwan and meet the regulations specified by the National Institute of Health Guide for the Care and Use of Laboratory Animals. (IACUC-19-222). Male *Wistar* rats were sourced from BioLASCO Co., Ltd., Taipei, Taiwan. 

### 2.3. Establishment of NP

Partial sciatic nerve transection (PSNT) surgery was performed by following our previous protocols [31,33,34]. The sciatic nerve from the left leg of rats was gently exposed, and a prolene 7-0 needle was inserted carefully and semi-transected, in an intracranial direction. In the sham group of animals, the sciatic nerve was left intact and sutured without transection. Seven days following the surgery, changes in paw withdrawal thresholds to heat and tactile stimuli were recorded in sham and PSNT rats. Tactile sensitivity was tested before heat sensitivity with a one-hour intermission between the tests from day 1 to day 14. Rats with motor deficits or those that did not develop NP after PSNT were eliminated from further studies.

### 2.4. Intrathecal Catheterization and Osmotic Pump Infusion

Intrathecal catheters were installed under 2–2.5% isoflurane anesthesia. The cisternal tissue was gently sliced to implant a polyethylene catheter (PE 10 tubes, 8.0 cm) directed towards the spinal cord. The rostral end of the catheter was connected to the mini osmotic (Alzet 2001, Cupertino, CA, USA) for the vehicle (1 µL/h) or TEN (5 µg/1 µL/h) or TEN (5 µg) + EXE (0.1 µg)/1 µL/h and infused for 7 days. After the installation of the pump, all rats were sent back to an animal room and placed in separate cages, and maintained on a 12–h light/dark cycle with free access to food and water. For a single i.t injection, the catheter’s rostral end was pointed towards the top of the head and the muscle was stitched with sutures. Seven days post PSNT, rats are either treated with a single i.t injection of vehicle (5 µL) or TEN (5 µg/5 µL) or MOR (5 µg/5 µL) or naloxone (10 µg/5 µL). To check the involvement of opioid receptors, naloxone (10 µg/5 µL) was administered 15 min before the injection of TEN (5 µg/5 µL) or MOR (5 µg/5 µL). After drug administration, the catheters were flushed with 15 µL saline.

### 2.5. Behavior Test for Tactile Allodynia

The paw sensitivity was examined in the left hind paw through a Dynamic Plantar Aesthesiometer (Ugo Basile, Comerio, Italy). Animals are placed in polycarbonate chambers with a metal mesh at the bottom. Animals were allowed to acclimatize for 30 min before testing. The withdrawal threshold of the transected paw in PSNT rats or sham rats was measured by progressively increasing the weight from 1 to 50 g through a blunt metal filament (0.5 mm) directed towards the plantar region. Paw reflexes were measured thrice at 2-min intervals and the average was determined by limiting 50 g as a cut-off threshold to prevent injury.

### 2.6. Behavior Test for Thermal Hyperalgesia

Heat sensitivity was measured through a Hargreaves radiant heat apparatus (7371; Ugo Basile, Comerio, Italy; infrared setting 80). Rats are placed in individual polycarbonate chambers placed on the testing apparatus and acclimatized for 30 min. The apparatus with a movable infra-red heat source was continuously applied to the plantar surface of the left hind paw. Withdrawal reflex spontaneously stops the automated timer, and the readings were noted for all rats with 5–min intervals. The baseline upper limit was between 8 and 10 s in non-operated rats, so a 20–s was set as a cutoff time to prevent heat burns.

### 2.7. Tail Flick Assay

The tail-flick latency was measured by immersion of the animal tail in a hot water jug (52 ± 0.5 °C). The control rats displayed a tail-flick latency of approximately 2 ± 0.25 s, and a 10–s is set as a cut-off time to prevent injury.

### 2.8. Spinal Cord Sample Preparation and Western Blotting Analysis

After completion of experiments, animals were sacrificed using isoflurane anesthesia (Abbott Laboratories Ltd., Queenborough, Kent, UK), and the spinal cord samples were preserved at −80 °C. The spinal cords were placed in lysis buffer and subjected to ultra-sonication (Misonix, Inc., Farmingdale, NY, USA) and centrifugation at 15,000 RPM for half an hour at 4 °C. The supernatant was quantified by the Bradford protein assay. Protein denaturation was done by heating at 90 °C for 10 min and separated by 12% SDS-PAGE. The proteins were transmitted onto a polyvinylidene fluoride membrane (Pall, Ann Arbor, MI, USA) and kept for blocking with 5% skimmed milk in tris-buffered saline (0.05% Tween 20 in tris-buffered saline). The membrane was incubated overnight with the following primary antibodies at 4 °C; anti-GAPDH (1:2000; Santa Cruz Biotechnology, Santa Cruz, CA, USA) and mouse anti-GLP-1R (1:200; Abcam, Cambridge, UK). After overnight incubation, the samples were washed with Tris-buffered saline with 0.1% Tween 20. Then, the samples were incubated with horseradish peroxidase-conjugated anti-rabbit (1:5000; Leadgene Biomedical, Tainan, Taiwan) for three hours at room temperature and identified using Enhanced Chemiluminescence Western Blotting Kit (Advansta, Menlo Park, CA, USA). The intensity of blots was quantified using the ImageJ software.

### 2.9. Immunfluoroscence Studies

For the spinal cord immunofluorescence, rats were deeply anesthetized with isoflurane and decapitated. The dorsal quadrant of the lumbar spinal cord enlargement was detached and stored in paraformaldehyde for further usage. The samples were first fixed overnight with 4% formaldehyde followed by 30% sucrose cryopreservation overnight. The samples were then paraffinized and sectioned (10 µM). The slides were incubated for 12 h at 4 °C with primary antibody, FITC tagged Anti-GFAP Protein Antibody @ 1:100 (Alexa Fluor^®^ 488, MAB3402X, Merck, Darmstadt, Germany). The slides were then stained with DAPI (Sigma-Aldrich, St. Louis, USA) for 1 h and scanned with Pannoramic 205 FLASH II Slide scanner. Scanned images were procured by using case viewer software. Quantitative analyses were performed using the ImageJ software.

### 2.10. Experimental Protocol

To evaluate the analgesic effects of Ten and Mor, five groups consisting of six rats per group were used. Group I: sham surgery rats. Rats were not subjected to nerve transection and the left sciatic nerve was gently exposed and muscle and skin are sutured. Group II: PSNT-vehicle group (nerve-transected group infused with saline). Group III: PSNT-TEN group (nerve-transected group infused with teneligliptin). Group IV: PSNT-TEN + EXE group (nerve-transected group infused with teneligliptin + Exendin-3 (9–39) amide). Group IV: PSNT-Mor (nerve-transected group infused with Morphine). The animal behavior test was performed on Day -1 (Pre-surgery), Day 7 (7 days post-surgery), and Day 8, day 10, Day 12, and Day 14. After the completion of behavioral testing on Day 14, the animals were sacrificed and the spinal dorsal horn was collected for western blotting and immunofluorescence assays.

To evaluate the behavioral effects after pre-treatment with opioid antagonist Naloxone, seven groups of six rats per group were used. Group I: sham surgery rats. Rats were not subjected to nerve transection and the left sciatic nerve was gently exposed and muscle and skin are sutured Group II: Group II: PSNT-vehicle group (nerve-transected group injected with saline alone) Group III: PSNT-TEN group (nerve-transected group injected with teneligliptin). Group IV: PSNT-NAL + TEN (nerve-transected group injected with naloxone 15min before teneligliptin injection). Group V: PSNT-NAL (nerve-transected group injected with naloxone alone). Group VI: PSNT-MOR group (nerve-transected group injected with morphine alone). Group VII: PSNT-NAL + MOR (nerve-transected group injected with naloxone 15min before morphine injection). One hour after drug administration, the behavioral tests were performed.

### 2.11. Statistical Analysis

The data are expressed as the mean ± SD. All graphical representations and statistical calculations were aided by GraphPad Prism version 6.01. Two-way ANOVA, Tukey’s multiple comparisons test, and Student’s *t*-test were used to analyze the statistical significance.

## 3. Results

### 3.1. TEN Has Mild Analgesic Effects against Acute Pain 

**Figure 1:**
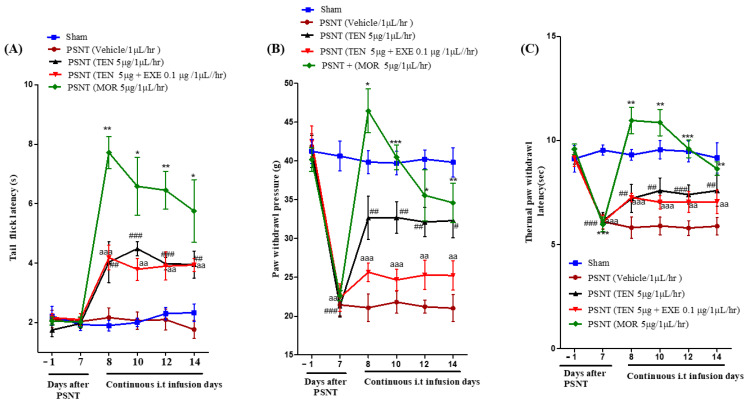
(**A**) Antinociceptive effects of TEN against acute pain. Tail−flick latencies were measured in sham rats and PSNT rats on days −1 (baseline before osmotic pump installation in PSNT rats) and day 7 after nerve transection. On day 7 osmotic pump is installed after baseline measurement and tail-flick latency was measure on days 8, 10, 12, and 14 by continuous i.t. infusion of vehicle (1 µL/h), TEN (5 μg/1 μL/h), TEN (5 μg) + EXE (0.1 μg)/1 μL/h, and MOR (5 μg/1 μL/h). (**B**) Effect of TEN continuous infusions on mechanical paw withdrawal pressure in PSNT rats. Paw withdrawal threshold against mechanical stimuli was measured in sham rats and PSNT rats on day −1 (Baseline before osmotic pump installation in PSNT rats), and on day 7 after nerve transection. On day 7 osmotic pump is installed after baseline measurement and paw withdrawal pressure was measured on days 8, 10, 12, and 14 by continuous i.t. infusion of vehicle (1 µL/h), TEN (5 μg/1 μL/h), TEN (5 μg) + EXE (0.1 μg)/1 μL/h, and MOR (5 μg/1 μL/h). (**C**) Effect of TEN continuous infusions on thermal paw withdrawal latency in PSNT rats. Paw withdrawal threshold against infra-red heat source is measured in sham rats and PSNT rats on days −1 (Baseline before osmotic pump installation in PSNT rats), and on day 7 after nerve transection. On day 7 osmotic pump is installed after baseline measurement and paw withdrawal latency was measured on days 8, 10, 12, and 14 by continuous i.t. infusion of vehicle (1 µL/h), TEN (5 μg/1 μL/h), TEN (5 μg) + EXE (0.1 μg)/1 μL/h and MOR (5 μg/1 μL/h). * Denotes statistical difference b/n sham group vs. PSNT (MOR), # denotes significant difference b/n PSNT (TEN) vs. PSNT (MOR), and “a” denotes significant difference b/n PSNT (TEN) vs. PSNT (MOR), * > 0.05, **/##/aa > 0.01, and ***/###/aaa > 0.001, (*n* = 6 animals per group).

Intrathecal infusion of TEN at a dose of 5 µg/µL/h resulted in a slight increase in the tail-flick latency. However, compared to MOR treated group, the analgesic effects were mild (Figure 1A black curve). Co-infusion of 0.1 µg GLP-1 antagonist EXE did not reverse TEN-induced acute antinociception in PSNT rats (Figure 1A red curve). 

### 3.2. PSNT Induces Tactile Allodynia and Thermal Hyperalgesia and TEN Alleviated PSNT Induced Thermal Hyperalgesia and Mechanical Allodynia

The paw withdrawal threshold to mechanical stimulus decreased from day 1–7 in PSNT rats similar to our previous reports (Figure 1B Black, red and brown curves) in comparison to sham surgery rats (Figure 1B Blue curve) [35]. In addition, paw withdrawal threshold to infra-red generated heat was significantly reduced from day 1–7 in PSNT rats (Figure 1C Black, red and brown curves) compared to sham surgery rats (Figure 1C Blue curve). The spinal infusion of TEN at a rate of 5 µg/1 µL/h showed a clear reduction in tactile allodynia and thermal hyperalgesia (Figure 1B,C black curves) from the initial day of infusion (day 8) when compared to vehicle-treated groups (Figure 1B,C). In contrast co-infusion of 0.1 µg GLP-1 antagonist, EXE partially reversed TEN analgesic effects on allodynia but not hyperalgesia in PSNT rats (Figure 1B,C red curves). Morphine (positive control) showed potential analgesic effects on day-7 but there is a significant decrease in antinociception on day 14 in both allodynia and hyperalgesia due to the opioid-induced tolerance.

### 3.3. Intrathecal Naloxone Pre-Treatment Antagonized MOR Analgesic Effect but Not TEN Induced Analgesia on Both Acute Pain and NP

**Figure 2:**
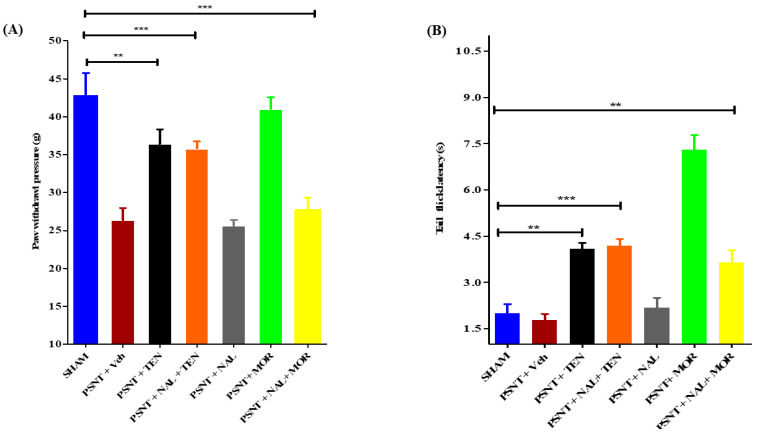
Effect of naloxone pre-treatment on TEN’s antinociceptive effects in PSNT rats. The effect of intrathecal opioid antagonist pretreatment on (**A**) Mechanical allodynia and (**B**) tail-flick latency are evaluated by administering a single i.t injection of naloxone (10 μg) 15 min before the i.t injection of TEN (5 µg/5 µL) or MOR (5 µg/5 µL). The effects of antinociception are compared with the sham group and the rats treated with a single i.t injection of vehicle (5 µL) or naloxone (10 µg/5 µL) or TEN (5 µg/5 µL) or MOR (5 µg/5 µL) in PSNT rats. * denotes a statistically significant difference between sham vs. PSNT + TEN, sham vs. PSNT + NAL + TEN and sham vs. PSNT + NAL + MOR. ** *p* < 0.01; *** *p* < 0.001, (*n* = 6 animals per group).

An i.t single injection of naloxone (10 µg) before TEN (5 µg) or MOR (5 µg) administration abolished the anti-allodynic effects and the antinociceptive action in tail-flick latency of MOR (Figure 2A,B yellow bars) but failed to reduce the analgesic effects of TEN (Figure 2A,B orange bars). Administration of naloxone (10 µg) alone did not have any significant analgesic effects in both mechanical allodynia and tail-flick latency (Figure 2A,B grey bars) when compared to sham and vehicle-treated PSNT rats. The naloxone dose chosen was based on previously reported papers [36].

### 3.4. TEN Reverses the Decrease in Spinal GLP-1 Expression in the PSNT Rats

**Figure 3:**
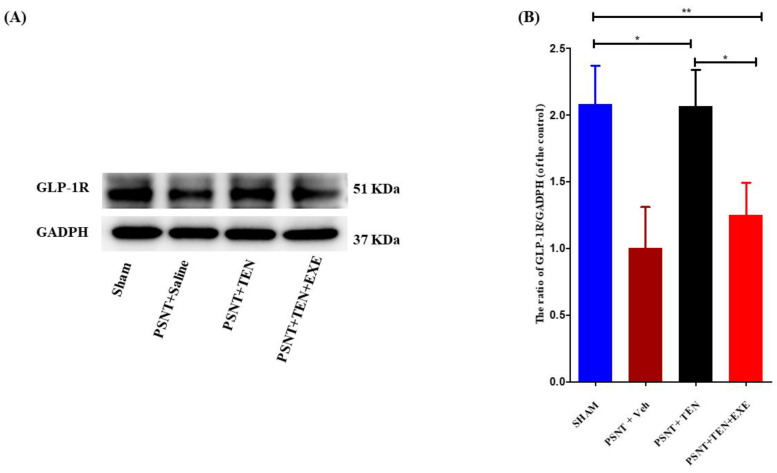
The expression and activation of GLP-1R by TEN. (**A**) Western blot and (**B**) The quantitative analysis of GLP-1R in sham and PSNT rat models i.t infused with vehicle (1 µL/h) or TEN (5 μg/1 μL/h) or TEN (5 μg) + EXE (0.1 μg)/1 μL/h for 7 days. Asterisk denotes a statistically significant difference between sham vs. PSNT + TEN, sham vs. PSNT + TEN + EXE and PSNT + TEN vs. PSNT + TEN + EXE. * *p* < 0.05; ** *p* < 0.01, (*n* = 6 animals per group).

Western blot assays revealed that GLP-1R protein expression was significantly downregulated in the PSNT group, but the infusion of TEN restored the levels of GLP-1R expression in the spinal dorsal horn (Figure 3A,B). Additionally. Co-infusion of GLP-1R antagonist significantly reduced the expression of GLP-1R compared to TEN infused rats (Figure 3A,B).

### 3.5. TEN Inhibits Spinal GFAP Activation in PSNT Rats

**Figure 4:**
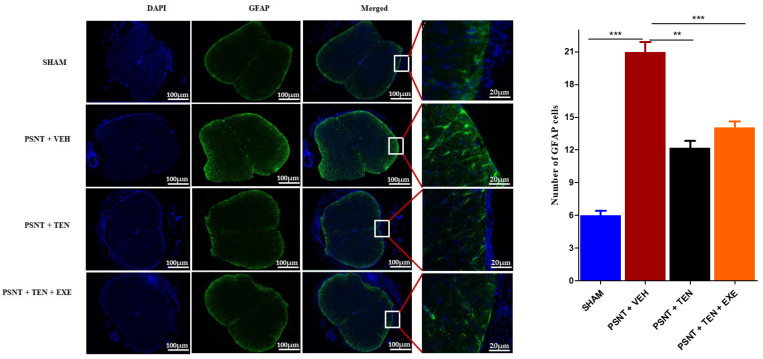
Effects of TEN administration on PSNT-induced glial fibrillary acidic protein (GFAP, astrocytic marker) expression in rat spinal cords. On day 7 after, spinal cord sections were fixed and single-labeled with fluorescein isothiocyanate-labeled anti-GFAP antibodies (green; astrocytes) and the nuclei were counterstained with 4′,6-diamidino-2-phenylindole (DAPI) (blue), then images were captured and merged by fluorescence microscopy. The sections were from sham rats, PSNT + vehicle infused rats, and PSNT + TEN infused rats. The green fibers indicate activated astrocytes. Asterisk denotes a statistically significant difference between sham vs. PSNT + Veh, PSNT + Veh vs. PSNT + TEN, and PSNT + TEN vs. PSNT + TEN + EXE. ** *p* < 0.01; *** *p* < 0.001, (*n* = 6 animals per group).

Astrocyte activation is an important indicator of oxidative stress in NP. Immunofluorescence results showed that the numbers of GFAP positive cells in the spinal dorsal horn were significantly higher in the PSNT group than in the vehicle-treated animals. However, TEN infusion significantly suppressed the number of GFAP positive cells in the spinal cord dorsal quadrant portion (Figure 4 black bar).

## 4. Discussions

The analgesic potential of DPP-4i TEN has not been previously investigated in animal models for acute and neuropathic pain. So, in the current report, we first investigated the antinociceptive effects of TEN in acute pain using the tail-flick assay. Second, we investigated the role of TEN in alleviating PSNT-induced NP by measuring paw withdrawal threshold against the mechanical and thermal stimulus. TEN infusion at a dose of 5 µg/µL/h did not have sufficient analgesic effect in tail-flick response (30%) compared to positive control MOR and co-infusion with antagonist EXE 0.1 µg/µL/h did not have any influence on TEN analgesia. However, unlike MOR, the partial analgesic effects from TEN remained stable for 7 days without any reduction in analgesic effects. This could be due to the slight analgesic tolerance from MOR continuous infusion. Previous studies mentioned that only therapeutics having >70% analgesia are considered to possess sufficient analgesic properties in acute pain [37]. The poor aqueous solubility of TEN is a limiting step to escalate the dose in our study, and increasing the concentration of DMSO is known to interfere with analgesia [38]. Thus, the effect of higher doses of TEN on acute antinociception might need more investigation through an oral or intraperitoneal route of administration. TEN i.t infusion at 5 µg/µL/h did not induce any motor blockade as the animals displayed normal locomotory activities. Nevertheless, our results can be corroborated with the modest analgesic effects of orally administered TEN against thermal pain in human subjects [14]. In addition, partial analgesic effects of other DPP4i have been reported previously [39]. 

To examine TEN’s effect against NP, we have developed a PSNT model used in our previous studies [34]. PSNT is a well-known model for NP. Along with PSNT, various other animal models were developed to study NP such as spared nerve injury (SNI), Sciatic chronic constriction injury (CCI), spinal nerve ligation (SNL), partial sciatic nerve ligation (PSNL). Nevertheless, SNL is vulnerable to microbial infections and is known to induce motor deficit [40,41]. Whereas the difference in ligature snugness can impact pain perception in the CCI model [40,41]. We have used a PSNT model, as it induces nerve damage without epineural inflammation [40]. Our model showed that transection of the sciatic nerve developed mechanical allodynia and hyperalgesia in the left leg of rats by day 7. Allodynia and Hyperalgesia remained stable after day 7 and the results are in line with our previous reports [35]. In our results, rats infused with TEN showed a clear reduction in both mechanical and thermal hypersensitivity from the initial day of treatment to the last day, whereas co-infusion with GLP-1R antagonist EXE slightly reduced the anti-allodynic and anti-hyperalgesic effects of TEN confirming that TEN could also induce antinociception through a GLP-1R independent mechanism. To investigate the involvement of opioid receptors, we blocked opioid receptors with naloxone and then TEN or MOR were administered. The antinociceptive action of TEN was not antagonized by pretreatment with naloxone but completely antagonized the antinociceptive effects of MOR, suggesting opioid independent analgesic effects.

Recently, the GLP-1R signaling pathway has emerged as a potential therapeutic target in cardiovascular and neurodegenerative diseases [42,43]. Several studies have focused on understanding the role of GLP-1/GLP-1R at the spinal level in neuroprotection and the regulation of antinociception [19,44]. GLP-1 is known to exert neuroprotection through suppression of neuroinflammatory, alleviation of oxidative stress, improving autophagy and apoptosis attenuation [45]. However, endogenous GLP-1 is prone to degradation from DPP-4, with a very short half-life. TEN is a selective DPP-4i, recently approved for the treatment of type-2 diabetes with a half-life of 24 h [46,47]. Apart from glycemic control, TEN is efficient in enhancing sudomotor function and reduce vascular inflammation in diabetic patients [48]. TEN is reported to enhance the beneficial effects of GLP-1 in cells and humans [18,49]. Our results demonstrated that TEN displayed great potential in alleviating NP in PSNT rats through a GLP-1R independent mechanism. Though several reports have shown the neuroprotective effects of TEN by the upregulation of GLP-1R in neurodegenerative diseases, our findings show that TEN can also induce antinociception and neuroprotection through GLP-1R independent mechanisms [29,50,51,52,53,54].

Recent research evidence suggests that astroglia play an important role in the pathogenesis of NP and inflammation. There are several reports on the anti-inflammatory effects of TEN [55,56,57,58]. A relationship has been established between astrocyte activation and increased hypersensitivity [59]. In addition, astrogliosis (revealed by enhanced GFAP expression) has been reported consistently in various chronic pain conditions such as SCI, nerve trauma, and long-term opioid exposure [60,61,62]. However, to the best of our knowledge, there are no studies that have investigated the role of TEN on astrocyte activation in NP model. In the present study, TEN administration significantly decreased astrocyte activation compared to the vehicle-treated group. From the results, we can assume that TEN’s analgesic effects might associated with the suppression of spinal astrocyte activation thereby preventing neuroinflammation and pain. 

In conclusion, our results have provided a piece of combined evidence to demonstrate that TEN alleviates PSNT-induced NP through the suppression of spinal astroglial cells. Our work lays a basement for prospective clinical translation of TEN for the effective management of NP in diseases involving the central nervous system (e.g., stroke, multiple sclerosis, tumor, and spinal cord injury) without serious side effects. Further investigation is necessary to confirm the possible role of GLP-1R in the antinociception of TEN through gene knockout animal models of NP.

## Data Availability

The data presented in this study are available within the article.
